# The *Arabidopsis* Transcription Factor CDF3 Is Involved in Nitrogen Responses and Improves Nitrogen Use Efficiency in Tomato

**DOI:** 10.3389/fpls.2020.601558

**Published:** 2020-11-24

**Authors:** José Domínguez-Figueroa, Laura Carrillo, Begoña Renau-Morata, Lu Yang, Rosa-V Molina, Daniel Marino, Javier Canales, Martin Weih, Jesús Vicente-Carbajosa, Sergio G. Nebauer, Joaquín Medina

**Affiliations:** ^1^Centro de Biotecnología y Genómica de Plantas, Universidad Politécnica de Madrid (UPM) – Instituto Nacional de Investigación y Tecnología Agraria y Alimentaria (INIA), Madrid, Spain; ^2^Departamento de Producción Vegetal, Universitat Politécnica de Valencia, Valencia, Spain; ^3^Department of Plant Biology and Ecology, University of the Basque Country (UPV/EHU), Bilbao, Spain; ^4^Instituto de Bioquímica y Microbiología, Facultad de Ciencias, Universidad Austral de Chile, Valdivia, Chile; ^5^ANID–Millennium Science Initiative Program-Millennium Institute for Integrative Biology (iBio), Santiago, Chile; ^6^Department of Crop Production Ecology, Swedish University of Agricultural Sciences, Uppsala, Sweden

**Keywords:** CDF, nitrate, tomato, photosynthesis, crop yield, C/N metabolism, transcriptome

## Abstract

Nitrate is an essential macronutrient and a signal molecule that regulates the expression of multiple genes involved in plant growth and development. Here, we describe the participation of *Arabidopsis* DNA binding with one finger (DOF) transcription factor CDF3 in nitrate responses and shows that *CDF3* gene is induced under nitrate starvation. Moreover, knockout *cdf3* mutant plants exhibit nitrate-dependent lateral and primary root modifications, whereas *CDF3* overexpression plants show increased biomass and enhanced root development under both nitrogen poor and rich conditions. Expression analyses of *35S::CDF3* lines reveled that CDF3 regulates the expression of an important set of nitrate responsive genes including, *glutamine synthetase-1*, *glutamate synthase-2*, *nitrate reductase-1*, and nitrate transporters *NRT2.1*, *NRT2.4*, and *NRT2.5* as well as carbon assimilation genes like *PK1* and *PEPC1* in response to N availability. Consistently, metabolite profiling disclosed that the total amount of key N metabolites like glutamate, glutamine, and asparagine were higher in *CDF3*-overexpressing plants, but lower in *cdf3-1* in N limiting conditions. Moreover, overexpression of *CDF3* in tomato increased N accumulation and yield efficiency under both optimum and limiting N supply. These results highlight CDF3 as an important regulatory factor for the nitrate response, and its potential for improving N use efficiency in crops.

## Introduction

Nitrogen (N) is an essential macronutrient and its availability in soil is a crucial factor for plant growth, distribution, and crop productivity. Nitrate (NO_3_^−^) is the main source of inorganic nitrogen for land plants (Krapp et al., 2014; Vidal et al., 2014). In addition, NO_3_^−^ plays a key function as a signaling molecule in many aspects of plant metabolism and developmental processes like the ones involved in seed germination, root, and shoot development and senescence (Scheible et al., 1997; Stitt, 1999; Crawford and Forde, 2002; Little et al., 2005; Vidal and Gutierrez, 2008). Global expression analyses of *Arabidopsis* plants under different N treatments revealed changes in expression levels of a large set of genes, including those involved in N transport and assimilation. Moreover, N supply promotes changes in the expression of genes involved in abiotic stress responses, carbon (C) metabolism, regulation of C/N balance, and signaling transduction, like transcription factors (TFs), kinases, and phosphatases (Gutierrez et al., 2007). Thus, several TFs implicated in the regulation of gene expression and signaling by NO_3_^−^ have been identified so far, including NIN Like protein 7, NLP7 (Castaings et al., 2009; Alvarez et al., 2020), NLP8 (Yan et al., 2016); TGA1 and TGA4 (Alvarez et al., 2014, 2019), SPL9 (Krouk et al., 2010), LBD37, LBD38, and LBD39 (Rubin et al., 2009), bZIP1 (Obertello et al., 2010), TCP20 (Guan et al., 2014), and ANR1 (Zhang and Forde, 1998). However, our understanding of the diverse regulatory pathways and the molecular mechanisms by which the different TFs modulate NO_3_^−^ responses is still limited.

DNA binding with one finger (DOF) TFs are a group of plant specific proteins that contain a highly conserved DNA binding domain of 52 amino acids, with a C2-C2 structure that binds to a 5-T/AAAAG-3 DNA sequence motif (Yanagisawa and Schmidt, 1999). Different reports showed that DOF proteins are involved in a wide range of developmental processes such as root growth, seed development, and flowering time (Yanagisawa, 2002; Noguero et al., 2013; Rueda-Lopez et al., 2017). In addition, maize ZmDOF1 and ZmDOF2 have been also implicated in nitrogen assimilation and C/N balance (Yanagisawa and Sheen, 1998; Yanagisawa, 2004; Peña et al., 2017). The overexpression of ZmDOF1 in *Arabidopsis* and rice enhanced the expression of genes involved in N assimilation like *glutamine synthetase (GS)* and *glutamate synthase* and genes encoding enzymes for carbon skeleton production like *C4-phosphoenol pyruvate carboxylase* (*PEPC*) and *pyruvate kinase1* (*PK1*). Moreover, ZmDOF1 overexpressing lines also showed increased amounts of amino acids, especially glutamine and an elevation in the nitrogen content (Yanagisawa, 2004). Most notably, Arabidopsis and rice plants expressing Dof1 showed better growth under low-nitrogen conditions (Yanagisawa, 2004; Kurai et al., 2011; Peña et al., 2017). Besides, a group of DOF factors, whose transcripts oscillate under constant light conditions named Cycling Dof Factors (CDF1-5; Imaizumi et al., 2005; Fornara et al., 2009), play a central role in the photoperiodic pathway controlling flowering-time in *Arabidopsis*. Moreover, *Arabidopsis* and tomato CDFs play additional functions in abiotic stress responses (Corrales et al., 2017; Renau-Morata et al., 2017, 2020a). In fact, both *Arabidopsis* and tomato (*AtCDFs* and *SlCDFs*) are differentially regulated by abiotic stress conditions like dehydration, osmotic, salt, heat stress, and cold stress (Corrales et al., 2014, 2017). Previously, we reported that the *Arabidopsis* KO mutant *cdf3-1* is more sensitive to drought and low temperature stress, whereas *CDF3* overexpression enhances the tolerance of transgenic plants to drought, cold, and osmotic stress and promotes late flowering (Corrales et al., 2017). Similar results have been also reported for tomato SlCDFs. In fact, overexpression of tomato *SlCDF1* and *SlCDF3* genes in *Arabidopsis* (Corrales et al., 2017) and *AtCDF3* and *SlCDF3* in tomato (Renau-Morata et al., 2017) increased drought and salt tolerance, and salt stress resistance, respectively. In addition, recent studies point to an involvement of CDFs in the responses to nitrogen. Network analyses using a time-based machine learning method applied to 2,174 dynamic N-responsive genes identified 155 regulators, including among them TFs previously validated in the N response (e.g., NLP7/8, TGA1/4, NAC4, HRS1, SNZ, and LBD37). This study also showed that those TFs are connected with additional second layer of TFs, which includes CDF1 (Varala et al., 2018). In addition, it is reported that master factor NLP7 targets multiple TFs including LBD37, LBD38, TGA4, HAP2C, NAC096, and CDF1 using the TARGET system (Alvarez et al., 2020), suggesting that CDF1 is a component of the regulatory network involved in N responses. In addition, we previously reported that tomato and *Arabidopsis* plants overexpressing the *AtCDF3* gene, the closest homolog of CDF1, exhibited changes in primary metabolism, with increased amounts of amino acids like glutamine, asparagine and GABA, and higher sucrose contents (Corrales et al., 2017; Renau-Morata et al., 2017). Moreover, the tomato *CDF3* overexpressor lines also showed higher yield and modified fruit amino acid and sugar content (Renau-Morata et al., 2017). These results suggested that CDF3 might play a role in nitrogen assimilation.

In the present work, we explored the function of CDF3 in N nutrition in *Arabidopsis* and evaluated the impact of its overexpression in tomato on N accumulation and use efficiency. We provide functional evidence that CDF3 is involved in NO_3_^−^ assimilation. In addition, our data support that CDFs are potential candidate genes for improving nutrient use efficiency in tomato.

## Materials and Methods

### Plant Materials and Growth Conditions

#### Arabidopsis thaliana

The *Arabidopsis thaliana* ecotype Columbia (Col-0) was used as wild type (WT). The *35S::CDF3* lines were previously described (Corrales et al., 2017). The *cdf3-1* and *cdf3-2* T-DNA insertion knockout mutants (GK-808605 and SAIL_434_09, respectively) were obtained from NASC. For studies on seedlings, plants were grown on MS-modified basal salt media without N (M531, Phytotechnology Laboratories) containing 1% (w/v) sucrose and 0.8% (w/v) plant agar at 22°C under long day 16-h light/8-h dark photoperiod for the time indicated in the figure legends. The full N treatment plate contained 10 mM KNO_3_ (Kiba et al., 2012). For N-limiting conditions (1 mM KNO_3_), the ion equilibrium of the medium was ensured by replacing KNO_3_ by KCl. For the phenotypic analyses plant images were acquired and biomass measurements were obtained after 12 days of treatment. Furthermore, roots and shoots were dried at 72°C for 48 h to determine their dry weights.

To analyze the role of CDFs in nitrogen signaling, we measure *CDFs* mRNA level in time-course experiments after nitrate treatments. First, we analyzed the early response to nitrate addition. To do so, WT plants were grown for 7 days in 10 mM KNO_3_, starved for 3 days (0 mM KNO_3_), and at the beginning of the next light cycle re-supplied with 5 mM KNO_3_, or 5 mM KCl as a control, for 0, 20 min, 2.5 h, or 8 h. We applied 5 mM KNO_3_ treatment, because it has been reported that this treatment elicit fast and robust responses to NO_3_^−^ in *Arabidopsis* (Krouk et al., 2010; Alvarez et al., 2014; Canales et al., 2014). Second, we assayed CDF response for N starvation. To do so, WT plants were grown for 7 days in 10 mM KNO_3_ and at the start of the light period transferred to a nutrient solution with 0 or 10 mM KNO_3_ for 0, 1, 3, and 6 days. Then, plants were harvested and immediately frozen in liquid N_2_.

#### Solanum lycopersicum

Tomato (*Solanum lycopersicum* L. cv. Moneymaker) plants overexpressing the *Arabidopsis CDF3* gene were previously as described in Renau-Morata et al. (2017). Two homozygous lines (L2 and L10) for the gene were selected for the present work and non-transformed Moneymaker plants were used as WT controls (C).

For phenotypic analyses in growth chamber (25/18°C and 16/8 h photoperiod), seeds were germinated in Petri dishes. After germination, seedlings were cultivated for 15 days in trays filled with vermiculite and fertilized with 1/2 strength Hoagland no. 2 (Hoagland and Arnon, 1950). Thirty-day-old plantlets were transferred to 1 L pots containing expanded clay balls (2–3 mm diameter; Arlita™, Spain). Plants were fertilized with 1/2 Hoagland no. 2 nutrient solution without N and supplemented with 8 or 4 mM NO_3_^−^, as N non-limiting and limiting, respectively (Wahle and Masiunas, 2003). The amount of other essential elements was maintained unaltered as described by Hoagland and Arnon (1950). Ten different plants for each genotype and experimental condition were used and physiological and biomass determinations were performed after 25 days.

For the greenhouse experiments, imbibed seeds were germinated on a moistened mixture of peat moss and sand in growth chambers at 25/18°C and a 16/8 h photoperiod. Thirty-days-old plantlets were transferred to 15 L pots that contained coconut coir fiber and grown for 6 months. Differential levels of N supply (8 and 4 mM nitrate) were applied as described previously for growth chamber experiments. Thirty plants were used per genotype and treatment.

### *In vitro* Root Morphology Analysis

For root morphology analyses, *Arabidopsis* seeds were surface sterilized, stratified at 4°C for 2 days and grown for 12 days on vertical plates in a chamber at 22°C/18°C under long day (16 /8 h, light/dark) conditions. MS-modified basal salt media without N (M531, Phytotechnology Laboratories) containing 1% (w/v) sucrose, 0.8% (w/v) plant agar, and supplemented with 10 or 0.1 mM KNO_3_, as described by Alvarez et al. (2019). To compensate the potassium balance in the N-limiting media, KCl in its appropriate molarity was added. Three replicates per genotype and condition were performed and six seeds were used in every replicate. Plates were photographed and plant fresh weight was measured. Root length and number of lateral root (LR) were estimated using Image J software. Three replicates per genotype and condition were performed and six seeds were used in every replicate. Biomass and root morphology parameters were measure as previously mentioned.

### Histochemical GUS Staining

For histochemical analyses, 4-day-old *Arabidopsis pCDF3::GUS* transgenic plants harboring a 1-kb promoter region fused to the *uidA* coding sequence (Corrales et al., 2017) were grown in N-free MS-modified basal salt media (M531, Phytotechnology Laboratories), containing 1% (w/v) sucrose, 0.8% (w/v) plant agar, and supplemented with 10 or 0 mM KNO_3_. GUS staining was performed as described by Jefferson et al. (1987).

### Gene Expression Analyses

For qRT-PCR expression analyses, total RNA was isolated from *35S::CDF3*, *cdf3-1* and WT seedlings that were grown in plates under 10 or 1 mM KNO_3_ supply as N-non-limiting and limiting conditions for 12 days. In the case of tomato, qRT-PCR expression analyses the total RNA was isolated from leaves of 55-day-old Moneymaker cv. and *35S::CDF3* tomato plants grown in growth chamber conditions under 8 and 4 mM N nitrogen supply. Total RNA was isolated by phenol/chloroform method, following Oñate-Sánchez and Vicente-Carbajosa (2008), and its quality and quantification were assayed using a NanoDrop 2000 (Thermo Scientific). cDNAs were obtained from 2 μg of RNA using oligo(dT)23 primers (Promega) and the Avian Myeloblastosis Virus Reverse Transcriptase (AMV RT; Promega) according to the manufacturer’s instructions. The primers used for PCR amplification in *Arabidopsis CDF3* (*At3G47500*) *GLU1* (*AT5G04140*), *GS1.1* (*AT5G37600*), *GS1.4* (*AT5G16570*), *GS2* (*AT5G35630*), *ASN1* (*AT3G47340*), *NIA1* (*AT1G77760*), *NRT2.1* (*AT1G08090*), *NRT2.4* (*AT5G60770*), *NRT2.5* (*AT1G12940*), *PK1* (*AT3G08730*), *PEPC1* (*AT3G08730*), and tomato *NR* (*Solyc011g01381*), *GAD2* (*Solyc11g011920*), and *GS2* (*Solyc04g014510*) genes are described in Supplementary Table S1. *UBIQUITIN21* (Czechowski et al., 2005) and *UBIQUITIN3* (Hoffman et al., 1991) from *A. thaliana* and *S. lycopersicum*, respectively, were used as reference genes. A LightCycler®480 System (Roche) was used for real-time PCR (5 min at 95°C, and 45 cycles of 95°C for 10 s, 60°C for 20 s, and 72°C for 30 s) using LightCycler®480 SYBR Green I Master (Roche). In order to analyze the melting dynamic of the amplified products, a final dissociation step was added (5 s at 95°C, 1 min at 65°C, continuous 97°C and 30 s at 40°C). Three independent samples were used and each reaction was performed in triplicate. The relative expression levels of target genes were calculated by the 2^−ΔΔCT^ method (Livak and Schmittgen, 2001), where ΔC_t_ is the difference in threshold cycle number (C_t_) for target gene and references genes.

### Amino Acid Quantification

To determine individual amino acids, 12-day-old control plants (Col-0) and the KO-mutant *cdf3-1*, and two independent *35S::CDF3* lines were grown in agar plates with the medium previously described, supplemented with 1 or 10 mM KNO_3_ as N limiting and non-limiting conditions, respectively, for 12 days. Extraction, manipulation, and mass spectrometric analysis of samples followed an adapted protocol described in Corrales et al. (2014). Protein content was determined as described by Sarasketa et al. (2014).

### Physiological and Metabolic Characterization of Tomato Lines

Net CO_2_ photosynthetic rate and total plant biomass were determined in WT (Moneymaker cv.) and *CDF3* overexpressing (lines L2 and L10) tomato plants after 25 days of differential N supply. Photosynthesis was measured using an LI-6400 infrared gas analyzer (LICOR Biosciences, Lincoln, USA) as described in Renau-Morata et al. (2017). Total soluble sugars, total α-amino acids, and starch content quantifications were performed as described in Monerri et al. (2011). The agronomic performance of the transgenic lines was assessed at the end of the experiment by measuring total yield (g fruits/plant) in the greenhouse as described in Renau-Morata et al. (2017). Total C and N content in reproductive and vegetative organs was measured with an elemental analyzer at the Ionomic service of CEBAS-CSIC (Murcia, Spain).

### Enzyme Activity Assays

For *Arabidopsis*, protein was extracted from 12 day-old seedlings, and the activity of glutamine synthetase (GS), nitrate reductase (NR), and glutamate dehydrogenase (GDH) enzymes were determined as described by Sarasketa et al. (2014). For tomato plants, protein extracts were obtained from leaves of 55-day-old plants. NR activity was determined as described by Calatayud et al. (2008).

### Determination of N Accumulation Efficiency and Its Components in Tomato

The N accumulation efficiency (NAE) and its components were calculated in tomato (Moneymaker cv) and *CDF3* overexpressing lines according to the method described previously (Weih, 2014; Weih et al., 2018). See Appendix S1 for details.

## Results

### Expression of *CDF3* Gene in Response to N Availability

In a previous work, we identified a set of five DOF TFs from group D (CDFs) in *Arabidopsis* and tomato that are differentially expressed in vegetative tissues in response to diverse environmental conditions like drought, salinity, or extreme temperatures (Corrales et al., 2014, 2017). In addition, we reported that the overexpression of *AtCDF3* and *SlCDF3* in both *Arabidopsis* and tomato, altered metabolism since several amino acids like GABA, proline, glutamine, and asparagine are accumulated among others, therefore suggesting that CDFs might play important functions in the control of N metabolism (Corrales et al., 2017; Renau-Morata et al., 2017). In addition, recent studies point to an involvement of CDF1, the closest CDF3 homolog, in the responses to nitrogen (Varala et al., 2018). Moreover, it is reported that NLP7, a master regulator of the nitrogen signaling pathway, targets multiple TFs including CDF1 (Alvarez et al., 2020), suggesting that CDF1 plays a role in the regulatory network involved in N responses. However, there is still a lack of information regarding the specific roles of CDF1 and CDF3 in N responses. In this context, the objective of this work was to assess the potential involvement of CDF3 in nitrogen signaling and plant responses to nitrogen availability.

**Figure 1:**
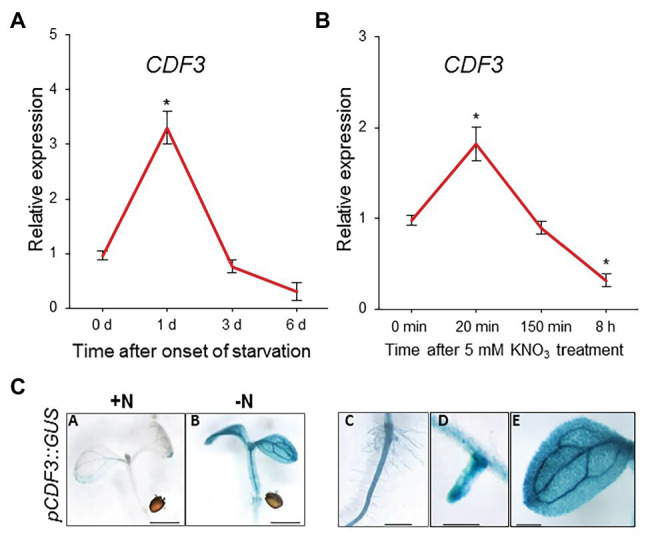
Expression patterns of *Arabidopsis CDF3* gene in response to nitrogen availability. qRT-PCR expression analyses of *Arabidopsis CDF3* gene in response to nitrogen availability. **(A)** To analyze the effect of starvation, the total RNA was extracted from plants grown in non-limiting N (10 mM KNO_3_) and thereafter transferred to 0 or 10 mM KNO_3_ for the indicated periods of time. Data are normalized to non-limiting N conditions. **(B)** To analyze the response to nitrate, 7-day-old plants were transferred, after 3 days of starvation, to MS medium supplemented with 5 mM KNO_3_ or KCl for the indicated periods of time. *UBIQUITIN21* gene was used as a reference gene. Data are normalized to KCl conditions. Data are means ± SE (*n* = 3). Asterisks indicate significant differences from control (*p* < 0.05); analysis of variance, followed by a Student-Newman-Keuls test **(C)** Histochemical localization of GUS activity of 7-day-old *pCDF3::GUS Arabidopsis* plants grown on plates supplemented with 10 mM (+N) or 0 mM KNO_3_ (−N) for 1 day (A–E). Bars indicate 300 μm. GUS staining of 7-day-old *pCDF3::GUS Arabidopsis* plants grown on N-depleted (−N) conditions (1d) showing expression of *CDF3* in (C) root hair zone in primary root, (D) emerging lateral root, and (E) close-up view of young leafs. Scale bars indicate 200 μm (C,D) and 1 mm (E). Photographs are representative of at least five independent experiments.

To investigate the possible role of CDF3 in nitrate responses, we first evaluated whether its expression is regulated by N availability. We studied the expression patterns of *CDF3* under N starvation or after a resupply of NO_3_^−^. In N starvation experiments, we performed qRT-PCR expression analyses using RNA isolated from 7-day-old WT plants that were initially grown with 10 mM NO_3_^−^ and then transferred to a nutrient solution without N. Expression levels were measured at 0, 1, 3, and 6 days. Under these conditions, the expression levels of *CDF3* increased showing the highest levels at 1 day after N starvation and decreased over the time (Figure 1A). We further analyzed the expression *CDF3* in response to N resupply treatment in a time course experiment. Plants (WT) were grown with 10 mM nitrate for 7 days, and then transferred to N depleted medium for 3 additional days. Afterward, plants were moved again to medium, containing 5 mM KNO_3_ or 5 mM KCl as a negative control for 0, 20 min, 2.5 h, and 8 h. We used 5 mM KNO_3_ treatment, because it has been shown by different research groups that this treatment elicit fast and robust responses to NO_3_^−^ in *Arabidopsis* (Krouk et al., 2010; Alvarez et al., 2014; Canales et al., 2014). As shown in Figure 1B, transcript levels of *CDF3* changed significantly in response to NO_3_^−^. The expression of *CDF3* was slight and transiently induced by NO_3_^−^ reaching a maximum level at 20 min, but repressed at longer times (8 h). These results indicate that the expression of *CDF3* is regulated by NO_3_^−^ availability.

In order to perform more detailed analyses of the spatial expression patterns of CDF3 in response to NO_3_^−^, we analyzed 7-day-old *pCDF3::GUS* plants grown under N-non-limiting conditions (10 mM N) and then transferred to a nutrient solution without N (1 day). Figure 1C shows that under N non-limiting conditions GUS staining was mainly detected in the vascular systems of roots, stems, and cotyledons (A). When *pCDF3::GUS* seedlings were grown under N depleted conditions (B–E), GUS staining was increased in emerged LR, root hairs, stems, and cotyledons, being especially strong in the vascular tissues (C–E). All these results indicate that *CDF3* gene is clearly expressed under N starvation conditions in tissues or cells that are involved in plant responses to N availability.

### Overexpression of *CDF3* Increases Plant Biomass Under Both N Limiting and Non-limiting Conditions

**Figure 2:**
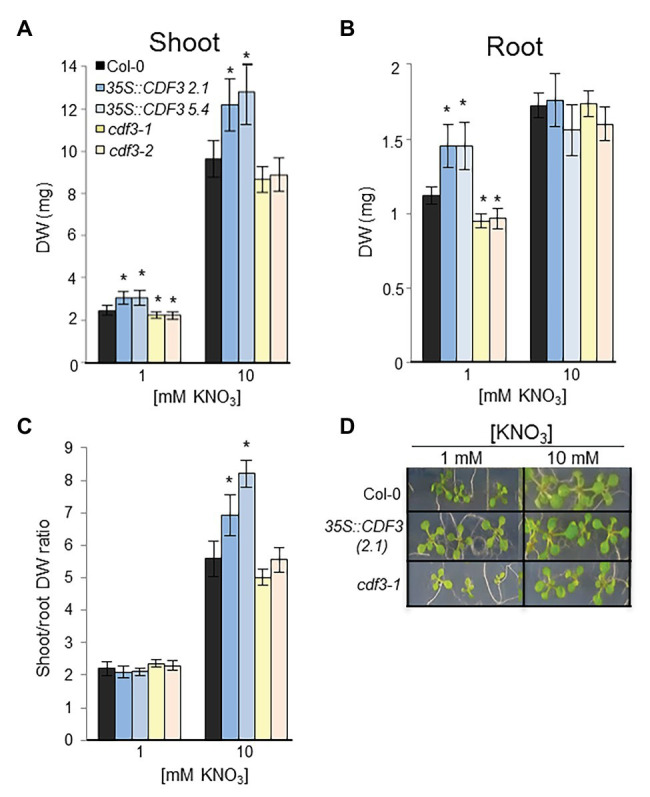
CDF3 effects on *Arabidopsis* plants growth under different N conditions. Phenotypes and biomass measurements of Col-0, *cdf3-1, cdf3-2*, and *CDF3* overexpressor plants (Lines 2.1 and 5.4) on MS medium containing different concentrations of potassium nitrate as sole nitrogen source. Biomass was measured as dry weight per plant. The photographs and biomass measurements were obtained after 12 days after treatment. **(A)** Shoot and **(B)** root dry weight (DW) of 12-day-old plants WT (Col-0), and *CDF3 gain- and loss-of-*function lines grown under different nitrate conditions (1 and 10 mM KNO_3_). Values are the mean ± SE of three independent replications each containing 20 plants per genotype. **(C)** Shoot-root DW ratio. Asterisks indicate significant differences compared with wild-type (Col-0; *p* < 0.05); analysis of variance Student-Newman-Keuls tests. **(D)** Representative pictures of the analyzed plants.

The results of the expression analyses were performed, suggested that CDF3 might play an important role in plant responses to NO_3_^−^. To further investigate this possibility, a phenotypic analysis of *CDF3* gain- and loss-of-function plants was done by analyzing their growth under both limiting and non-limiting N conditions (Figure 2). We analyzed a previously identified T-DNA insertion mutant *cdf3-1* (Fornara et al., 2009; Corrales et al., 2017) and a new mutant allele (*cdf3-2*; SAIL 434_G09) that we have identified with the T-DNA insertion site located at position 651 from the ATG (Supplementary Figure S1A). The disruption was verified by almost the absence of *CDF3* expression (Supplementary Figure S1B). In addition, two *CDF3* overexpressor *Arabidopsis* lines were included in the study (L2.1 and L5.4; Corrales et al., 2017). Plants were grown on MS medium supplemented with a range of N conditions including 1 and 10 mM KNO_3_, and plant biomass was evaluated after 12 days of growth. Under N limiting conditions (1 mM KNO_3_), *CDF3* overexpressor lines showed better performance compared to WT and *cdf3-1*, keeping healthy greener leaves and showing higher values of root biomass (Figures 2A,C). In contrast, *cdf3-1* and *cdf3-2* displayed lower size and values of shoot and root biomass than WT plants on medium containing 1 mM nitrate. On the other hand, under N non-limiting conditions (10 mM KNO_3_), *cdf3-1* and *cdf3-2* lines showed similar values of shoot and root biomass and water content (about 81%) than the WT. But, *CDF3* overexpressor plants exhibited significant higher values of shoot biomass than WT and *cdf3* lines. Consequently, the shoot to root DW weight ratio, an important parameter influenced by nutrient availability (Lawlor et al., 2001), was significantly higher for *CDF3*-overexpressing plants at 10 mM NO_3_^−^ conditions but slightly lower at limiting N supply (Figure 2C). However, *cdf3-1* and *cdf3-2* lines showed similar ratios than the WT at both N limiting and non-limiting conditions. All these data suggest that CDF3 might be involved in the adjustment of root and shoot growth in response to N availability.

### CDF3 Impact on Root Morphology

**Figure 3:**
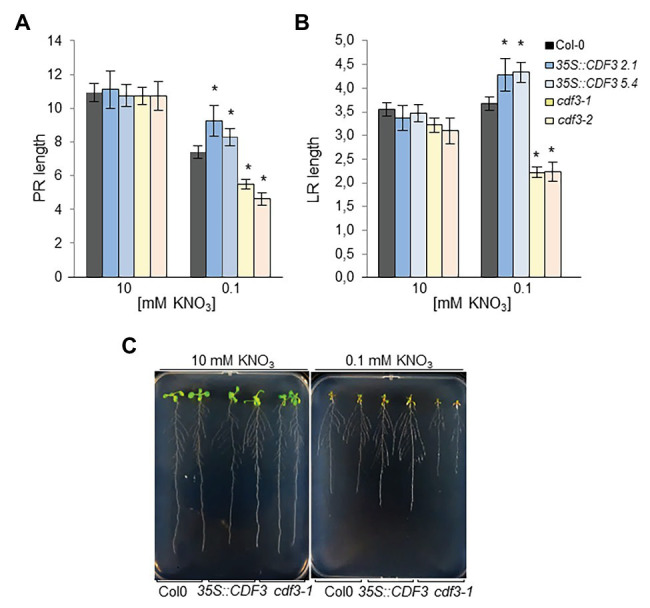
Root morphology of *CDF3* overexpressing, *cdf3* mutants, and WT plants under different N supply. Plants were grown on vertical plates with 10 or 0.1 mM KNO_3_ as sole N source, for 12 days. **(A)** Primary root (PR) length and **(B)** Lateral root (LR) length (cm) was estimated under different nitrate conditions. Data are means ± SE of three independent experiments with at least 20 plants each. Asterisks indicate significant differences compared with wild-type (Col-0; *p* < 0.05); analysis of variance, followed by Student-Newman-Keuls tests. **(C)** Representative pictures of the analyzed plants.

Nitrogen availability modulates gene expression affecting primary and LR growth and development (Little et al., 2005; Remans et al., 2006; Li et al., 2007; Canales et al., 2017). To further investigate the role of CDF3 in root morphology in relation to N availability, we analyzed the root system by estimating primary root (PR) and LR length in gain- and loss-of-function plants grown in 0.1 and 10 mM NO_3_^−^. These conditions have been previously shown to promote significant changes in *Arabidopsis* root morphology in root length assays in vertical plates in short-term experiments (Zhang and Forde, 1998; Varala et al., 2018; Alvarez et al., 2020). As shown in Figure 3A, under 10 mM NO_3_^−^ no significant differences in the main root length were found between gain- and loss-of-function lines and WT plants. In contrast, under 0.1 mM NO_3_^−^ supply, *35S::CDF3* plants showed moderate but significant higher values of relative PR length growth than WT, whereas *cdf3* plants exhibited lower values of PR relative growth. Notably, *cdf3* lines also showed lower values of LR length compared to WT plants (Figures 3B,C). In contrast, *CDF3* overexpression lines showed higher values of LR length compared to WT plants. More detailed analysis of the root system showed that both *CDF3*-overexpressing and *cdf3* plants showed no significant differences in LR density compared to the WT under both N limiting and non-limiting conditions, suggesting that CDF3 might play a more important role in PR and LR elongation than in branching (Supplementary Figure S2). Overall, these data indicate that CDF3 is involved in NO_3_^−^ modulation of root growth and development and overexpression of *CDF3* promotes root elongation.

### CDF3 Regulates the Expression of Genes Involved in Nitrogen Metabolism

**Figure 4:**
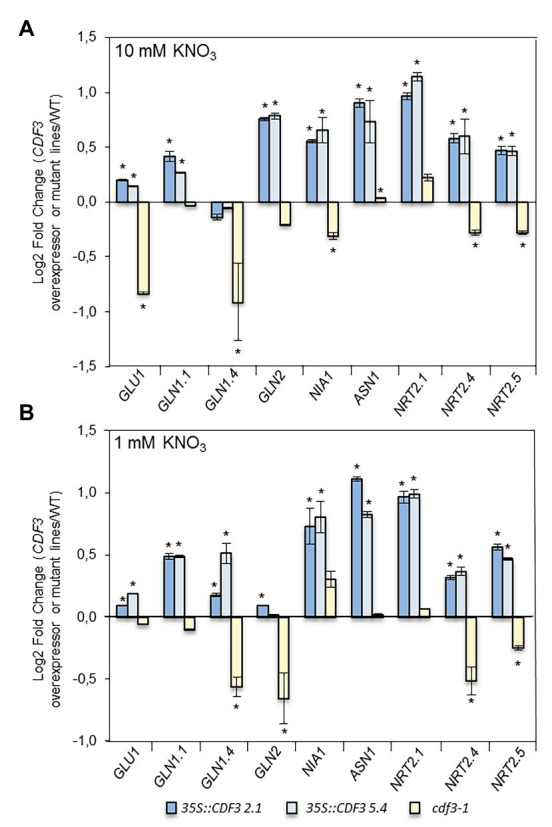
CDF3 regulates a set of genes related to nitrogen assimilation and transport. Expression analyses of N assimilation (*GLU1*, *GLN1.1*, *GLN1.4*, *GLN2*, *ASN1*, and *NIA1*) and nitrate transporter (*NRT2.1*, *NRT2.4*, and *NRT2.5*) genes by qRT-PCR in Col-0, *cdf3-1*, and *35S::CDF3* (L2.1 and L5.4) transgenic lines. Total RNA was extracted from 12-day-old plants grown on **(A)** MS medium containing 1 or 10 mM **(B)** KNO_3_ as sole nitrogen source. Log2 Fold Change (Log FC) values were generated by comparing the expression of genes at each N treatment of each line vs. the control (Col0) using the 2^−ΔΔCt^ method. *Arabidopsis UBIQUITIN21* gene was used as a reference gene. Data are means ± SE (*n* = 3). Asterisks indicate significant differences compared with Col-0; (*p* < 0.05) analysis of variance, followed by a Student-Newman-Keuls test.

To further gain insight into the molecular mechanisms underlying the observed NO_3_^−^ responses in *CDF3* gain- and loss-of-function lines, we performed detailed expression analyses of selected NO_3_^−^ responsive genes involved in N assimilation: *NIA1* encoding *nitrate reductase 1*, *glutamine synthetase 1.1* (*GLN1.1*), *GLN1.4* and *GLN2*, *asparagine synthetase 1* (*ASN1*), and *glutamate synthase 1* (*GLU1*). A set of genes encoding nitrate transporters *NRT2.1*, *NRT2.4*, and *NRT2.5* were also surveyed. We performed qRT-PCR using RNA isolated of *35S::CDF3*, *cdf3-1* and WT seedlings that were grown under 10 or 1 mM KNO_3_ supply as N non-limiting and limiting conditions for 12 days. As shown in Figure 4, in N non-limiting conditions, *CDF3* overexpressor lines showed higher levels of expression of genes involved in N assimilation: *GLU1*, *GLN1.1*, *GLN2*, *ASN1*, and *NIA1* compared to WT and *cdf3-1*. Remarkably, we observed that expression levels of all the nitrate transporters analyzed are higher in *35S::CDF3* lines compared with the WT (Figures 4A,B). In contrast, in the case of *cdf3-1* mutant, the genes analyzed display different expression patterns in N non-limiting conditions. While *GLN1.4*, *GLU1*, *NIA1*, *NRT2.4*, and *NRT2.5* genes exhibited lower expression levels than the WT, the rest of genes were analyzed and displayed similar levels of expression compared to the WT. Under N limiting conditions, most of the genes analyzed in *CDF3* overexpressor plants exhibited higher levels of expression than WT and *cdf3-1*. In the case of *cdf3-1* mutant, the majority of the genes was analyzed and exhibited similar transcript levels to the ones in the WT. However, the transcript levels of *GLN2*, *GLN1.4*, *NRT2.4*, and *NRT2.5* genes, which are involved in glutamine biosynthesis, nitrogen remobilization, and nitrate transport, respectively, especially in senescing organs (Masclaux-Daubresse et al., 2010; Kiba et al., 2012; Moison et al., 2018), were lower in *cdf3-1* than in control plants. This suggests that CDF3 might be involved not only in NO_3_^−^ assimilation but also in remobilization and transport in response to N availability.

**Figure 5:**
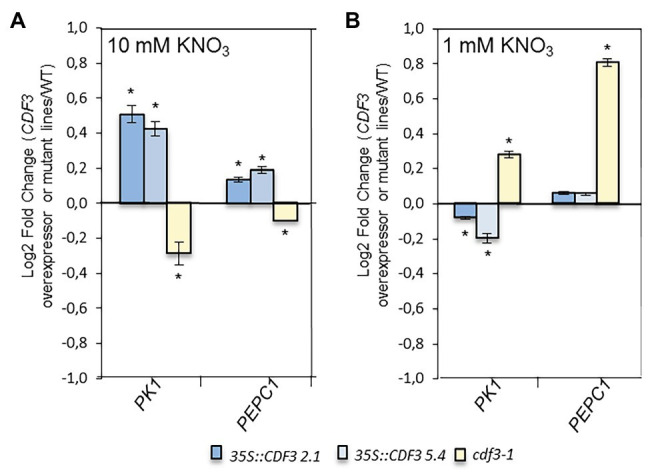
CDF3 regulates genes involved in carbon skeleton formation for N assimilation. Expression analyses by qRT-PCR of *PK1* and *PEPC1* genes in Col-0, *cdf3-1*, and *35S::CDF3* (L2.1 and L5.4) transgenic lines. Total RNA was extracted from 12-day-old plants grown on **(A)** MS medium containing 1 or 10 mM **(B)** KNO_3_ as sole nitrogen source. Log2 Fold Change (LogFC) values were generated by comparing the expression of genes at each N treatment of each line vs. the control (Col-0) using the 2^−ΔΔCt^ method. *Arabidopsis UBIQUITIN21* gene was used as a reference gene. Data are means ± SE (*n* = 3). Asterisks indicate significant differences compared with Col-0 (*p* < 0.05), analysis of variance, followed by a Student-Newman-Keuls test.

N assimilation requires both a source of inorganic nitrogen and a carbon skeleton for its incorporation, mainly 2-oxoglutarate (2-OG) that is produced through sequential reactions from photoassimilated carbohydrates. In addition, it has been shown that the maize TF DOF1 plays an important role in nitrogen assimilation (Yanagisawa, 2004) by regulating the expression of two genes encoding key enzymes involved in this process *PEPC1* and *PK1*. To further investigate the role of the *Arabidopsis* CDF3, we analyzed the expression of *PEPC1* and *PK1* genes in *CDF3* overexpressor, *cdf3-1* and WT plants. Under N non-limiting conditions, the expression levels of *PK1* and *PEPC1* are lower in the *cdf3-1* mutant and higher in both *CDF3* overexpressor lines compared with the WT control (Figure 5). In contrast, under N limiting conditions the expression of *PK1* and *PEPC1* are induced in the *cdf3-1* mutant line compared to the WT, and in the case of *PK1* slightly repressed in the *CDF3* overexpressor lines compared to the control. These results indicate that CDF3 can modulate the expression of *PK1* and *PEPC1* genes involved in the production of C skeletons for amino acid biosynthesis, depending on N availability.

### Enhanced N Assimilation in *CDF3*-Overexpressing *Arabidopsis* Plants

**Figure 6:**
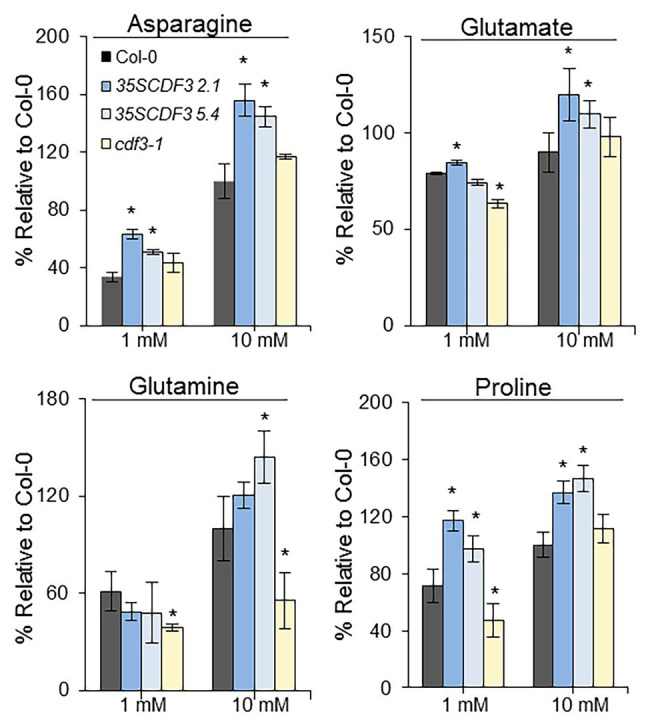
Effect of *CDF3* expression on individual amino acid levels. Relative quantities (% relative to wild type) of selected metabolites analyzed by gas chromatography-selected ion monitoring-mass of 12-day-old control plants (Col-0) and *cdf3-1*, and *35S::CDF3* (lines L2.1 and L5.4) grown in agar plates supplemented with 1 or 10 mM KNO_3_ (N limiting and non-limiting, respectively). Results are shown as means ± SE (*n* = 15). Similar results were obtained in five independent experiments; Asterisks indicate significant differences compared with control (*p* < 0.01); analysis of variance, followed by a Student-Newman-Keuls test.

To further investigate the observed differences in growth and gene expression of *CDF3* gain- and loss-of-function mutant lines under the different nitrate conditions, we analyzed the levels of major amino acids like glutamine, glutamate, proline, and asparagine, which are well-known markers of N assimilation. The metabolites were evaluated in 12-day-old *35S::CDF3*, *cdf3-1*, and WT plants that were grown under 1 or 10 mM nitrate supply, (N limiting and non-limiting conditions, respectively) as performed in growth and gene expression assays. As shown in Figure 6, under non-limiting N conditions, we found higher values of asparagine, glutamate, glutamine, and proline in *CDF3* overexpresor lines than in WT plants, whereas *cdf3-1* mutants showed similar values of proline, asparagine, and glutamate. On the other hand, under N limiting conditions, *cdf3-1* mutant showed lower levels of glutamate, glutamine, and proline amino acids compared to the WT. In contrast, *35S::CDF3* plants exhibited higher levels of asparagine and proline than control plants, but similar levels of glutamine and glutamate. Consistently, the activity of GS, NR, and GDH enzymes were higher in both *CDF3* overexpressor lines compared to the WT (Supplementary Figure S3), but lower in the *cdf3-1*, which might partly supported the glutamate and glutamine levels in the plants. Total protein content increased in the *CDF3*-overexpressing plants under 1 mM N but not under 10 mM N condition compared with WT plants, while in the case of *cdf3* mutant, the total protein content was significantly lower under N limiting conditions respect to WT plants but no change was observed at 10mM N (Supplementary Figure S4). Altogether, these observations might explain the amino acid contents detected in the different genotypes and suggest that CDF3 plays an important role in nitrate assimilation.

### *CDF3* Enhances Biomass Production and Crop Yield in Tomato Under Different N Supply

**Figure 7:**
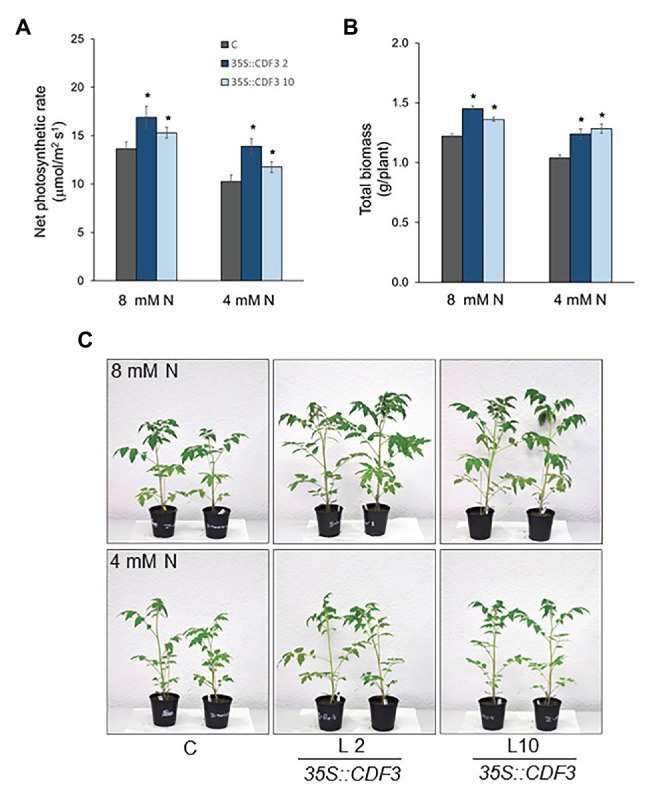
*CDF3* overexpression in tomato enhanced photosynthesis and biomass production under different N supply. Net photosynthetic rate **(A)** and total dry biomass **(B)** in *35S::CDF3* (L2 and L10; blue bars) and control (**C**; gray bars) Moneymaker tomato plants grown in hydroponic conditions under N non-limiting (8 mM N) and limiting (4 mM N) nitrogen supply for 25 days. Each value is the mean ± SE of 10 different determinations in different plants. Asterisks indicate significant differences compared with control (*p* < 0.05), analysis of variance, followed by a Student-Newman-Keuls test. **(C)** Representative pictures of the plants analyzed.

Since the data obtained in *Arabidopsis* suggested that CDF3 participates in the control N metabolism, we decided to assess whether *CDF3* can be used to improve N assimilation in an economically important crop plant like tomato. For this purpose, phenotypic and physiological analysis of the tomato (Moneymaker) plants overexpressing *CDF3* gene (lines L2 and L10) were studied when grown under different N supply. Tomato plantlets at the three-leaf-stage were grown for 25 days under contrasting 8 and 4 mM N supply, which in tomato have been shown as N non-limiting and limiting conditions, respectively (Wahle and Masiunas, 2003). Figure 7 shows that *CDF3* overexpressing plants exhibited improved growth with increased fresh weight compared to control plants under different N conditions. In addition, *CDF3* overexpressors displayed higher photosynthetic rates and biomass than control plants under both N conditions (Figures 7A–C; Supplementary Table S2). Consistently, *CDF3* overexpressing plants exhibited higher total sugar and total free amino acids content in leaves compared to WT (Supplementary Table S2). Moreover, expression analyses of the tomato genes *NR*, *GS2*, and *glutamate decarboxylase* (*GAD2*) involved in N assimilation and metabolism showed significant higher transcript levels in *CDF3* overexpressing plants than in the WT (Supplementary Figure S5). Besides, NR activity was higher in *35S::CDF3* plants than in control plants, both under N non-limiting (22.9 vs. 16.2 μmol NO_2_/m^2^s) and limiting (17.6 vs. 13.1 μmol NO_2_/m^2^s) conditions. These, results show that the overexpression of *CDF3* gene in tomato increases biomass production under both N treatments, and that this higher growth is sustained by a higher N and C assimilation.

**Figure 8:**
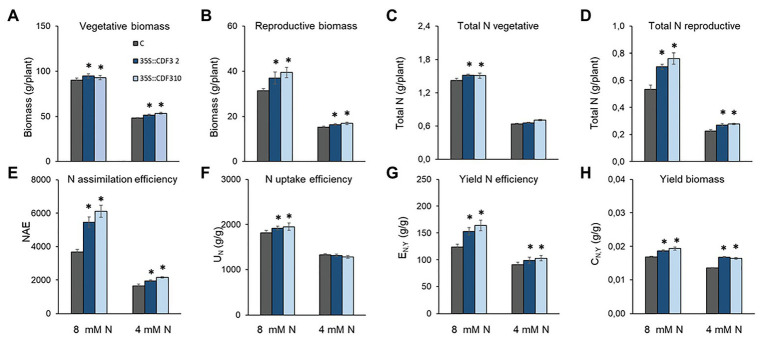
*CDF3* enhances nitrogen accumulation efficiency in tomato under contrasting N supply. Total biomass (g, DW/plant) in vegetative organs **(A)** and fruits **(B)**; total nitrogen content (g/plant) in vegetative organs **(C)** and fruits **(D)** of *35S::CDF3* (lines L2 and L10) and control Moneymaker plants **(C)**. Values are mean (±SE) of 10 different determinations in different 220-day-old plants grown under 4 mM (white bars) and 8 mM N (black bars) conditions. Values of **(E)** nitrogen accumulation efficiency (NAE) and its components: **(F)** N uptake efficiency (U_N_; g g^−1^), **(G)** yield-specific N efficiency (E_N,y_; g g^−1^) and **(H)** yield biomass (C_N,y_; g g^−1^). Asterisks indicate significant differences compared with control (*p* < 0.05); analysis of variance, followed by a Student-Newman-Keuls test.

Further, we investigated if the higher biomass production of the *35S::CDF3* tomato plants resulted in increased fruit production by measuring the number of fruits and fruit weight in *CDF3* overexpressor plants (L2 and L10 lines) and control plants. To do so, tomato plants were cultured in the greenhouse during 6 months under the different N treatments (8 and 4 mM N). Tomato *CDF3* overexpressing plants showed improved fruit yield under both N conditions (Figure 8B; Supplementary Figure S6). Specially, under 8 mM N fertilization, the increase in yield was related to both larger fruit size and number of fruits, whereas under low N conditions resulted from only larger fruit size, since fruit number remained similar between WT and *35S::CDF3* plants (Supplementary Figure S6). Remarkably, *35S::CDF3* plants showed higher values of dry biomass in fruits and vegetative tissues under both N conditions at the end of the culture (Figures 8A,B). The higher values of biomass under high N supply conditions are connected to higher N content in both vegetative and reproductive organs of *35S::CDF3* plants (Figures 8C,D). Under limited N supply, the N content was higher in the fruits, but similar in the vegetative organs of *35S::CDF3* and control plants. Notably, under 8 mM N supply, the values of percentage of total fruit biomass were higher in *35S::CDF3* than control plants (29.8 vs. 25.8% of control fruits, respectively), which might suggest an increased partition to the fruits in this genotype. Overall, these results suggest that CDF3 influenced N assimilation and biomass partition to the fruits in tomato, leading to increased yield both under optimal and limited N supply.

### The Overexpression of *CDF3* Increased NAE in Tomato

In order to determine the possible impact of CDF3 in NAE, we analyzed *CDF3* overexpressor (L2 and L10) and WT tomato plants, using the method proposed by Weih (2014). The results shown in Figure 8 indicated that the reported changes in C and N metabolism of plants overexpressing the *CDF3* gene were clearly reflected by corresponding changes in NAE and its components N uptake efficiency (U_N_), yield-specific N efficiency (E_N,y_), and fruit N content (C_N,y_; Appendix S1). Thus, under N non-limiting conditions *35S::CDF3* lines showed higher U_N_ than controls, indicating an increased capacity to take up and/or assimilate N during growth. Furthermore, the E_N,y_ and C_N,y_ were enhanced in *CDF3* overexpressing plants compared to the control (Figure 8) and contributed significantly to the increased NAE. The results are consistent with the increased biomass partitioning to the fruits and yield observed in *CDF3* overexpressor plants (Figures 8A–D). Under N limiting conditions, NAE was considerably lower compared to non-limiting N treatment in both genotypes, although *35S::CDF3* plants showed higher NAE than control plants due to higher values of E_N, Y_ and C_N,Y_ (Figure 8). Altogether, results indicate that the increased NAE of *35S::CDF3* plants is related to a greater efficiency of converting accumulated N into fruit biomass, allowing an increased partitioning of C and N compounds to these organs in both N treatments and increased N uptake efficiency only under optimum N supply.

## Discussion

In recent years, different reports showed that CDF TFs not only participate in the control of plant growth and development but also in the responses to different abiotic stresses (Corrales et al., 2014, 2017; Fornara et al., 2015; Renau-Morata et al., 2017, 2020a). In this study, we have identified that *Arabidopsis CDF3* expression is modulated by N availability. The presented data support that CDF3 plays a relevant role in relation to N supply, especially in nitrogen metabolism. CDF3 controls the expression of different nitrate-regulated genes involved in the acquisition, transport, and assimilation of N. Besides, this study also showed that the expression of *CDF3* gene in tomato enhances growth under both N-limiting and non-limiting conditions, resulting in higher yield and increased NAE and partition of C and N compounds to the fruits of *35S::CDF3* plants.

### New Function of CDF Factors in Nitrogen Responses

**Figure 9:**
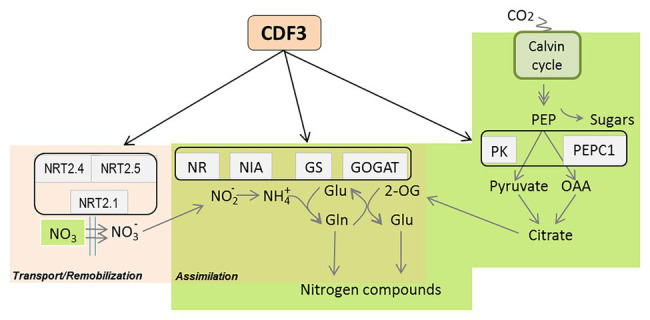
General scheme of CDF3 functions in nitrogen metabolism. CDF3 modulates the expression of the gene-modules involved in nitrogen assimilation and transport. PEP, phosphoenolpyruvate; PK, piruvate kinase; OAA, oxaloacetate; GOGAT, glutamate synthase; GS, glutamine synthetase; NIA/NR, nitrate/nitrite reductases; NRT2, nitrate transporter.

Previous works have shown that members of DOF TF family are implicated in N-regulated processes (Yanagisawa, 2004; Rueda-López et al., 2008; Renau-Morata et al., 2017, 2020a,b; Varala et al., 2018) motivating in this work a further comprehensive analysis of *CDF3* role in plant response to N availability. Detailed expression analyses by RT-qPCR showed that *CDF3* expression is transiently induced after 1 day of N starvation, indicating that it might participate in the assimilation/mobilization pathways. Further analyses of *gain- and loss*-*of-function* lines allowed us to confirm the role of CDF3 as an important factor in N-regulated processes. The overexpression of *CDF3* in *Arabidopsis* led to significant higher values of shoot and root biomass under N non-limiting and limiting conditions, respectively, together with a notable rise in the content of major amino acids and total protein and concurrently with an increase in enzyme activities of N metabolism (Figure 6; Supplementary Figures S3, S4). On the contrary, *cdf3* KO mutant plants had impaired N use ability and showed more severe N-deficient phenotypes including reduced growth, lower size and shoot, and root biomass reduction compared to WT, specially under N limiting conditions (Figures 2, 3). In sharp contrast to *cdf3-1* mutant, the overexpression of *CDF3* significantly enhanced the expression of important N-regulated genes involved N assimilation like *NIA1*, *ASN1*, *GLU*, *GLN2*, *GLN1.1*, and transport such as *NRT2.1*, *NRT2.4*, and *NTR2.5*, both under N limiting and non-limiting conditions (Figure 4). The increased transcript levels of this group of genes would be related to enhanced N assimilation as revealed in Figure 6 and Supplementary Figures S3, S4. Notably, the transcript levels of *GLN1.4*, *GLN2*, *NRT2.5*, and *NRT2.4* were found to be downregulated in the *cdf3* mutant under N limiting conditions. Interestingly, these groups of missregulated genes are involved in glutamine biosynthesis and N remobilization and NO_3_^−^ transport, mainly in senescing organs (Masclaux-Daubresse et al., 2010; Kiba et al., 2012; Moison et al., 2018). Using growth analyses of multiple mutant lines, it was established that NRT2.4 and NRT2.5 are required to support growth of N-starved adult plants by ensuring the efficient uptake of NO_3_^−^ collectively with NRT2.1 and NRT2.2 and by taking part in NO_3_^−^ loading into the phloem during N remobilization (Kiba et al., 2012; Lezhneva et al., 2014). All these observations support a multifaceted role of CDF3 in response to N limitation, modulating the expression of genes involved N assimilation and a set of NO_3_^−^ transporters, likely in different organs, and consequently would lead to enhanced metabolism and probably the transport/uptake of NO_3_^−^, to rapidly adapt to N availability and maintain plant N homeostasis. Supporting this hypothesis in silico expression analyses of *CDF3* using publicly available expression data (eFP browser),^1^ indicated that *CDF3* showed significant levels of expression in both root and shoot at the seedling stage and in senescing leaves, stems, and seeds in adult plants (Supplementary Figure S8). In Figure 9, a general scheme of the different group of genes regulated by CDF3 in *Arabidopsis* is proposed. In addition, the ectopic expression of *Arabidopsis CDF3* in tomato also promoted similar effects as in *Arabidopsis* (Figures 7, 8). We confirmed that tomato *CDF3* overexpressing plants exhibited improved growth and biomass under different N conditions together with higher levels of total free amino acids and N content compared to WT. In addition, the expression of *CDF3* in tomato also promoted enhanced expression of tomato genes involved in N metabolism like *NR*, *GS2* and *GAD2* (Supplementary Figure S5). Remarkably, the activity of NR enzyme was higher in both tomato and *Arabidopsis CDF3* overexpressing lines compared to WT plants (Supplementary Figure S3). All these results evidence a similar function of CDF3 in tomato and *Arabidopsis*, suggesting that this might be a conserved mechanism in plants. Overall, these data indicate that overexpression of *CDF3* can improve plant N use ability by regulating N metabolism and transport pathways depending on N supply.

### Implication of CDF3 in Root Morphology

Root and root hairs are organs that first sense N availability and display crucial morphological adaptations to N supply (Walch-Liu et al., 2005; Alvarez et al., 2014). Our results provide evidence of a new function for this transcription factor in nitrate regulation of gene expression and root developmental responses in *Arabidopsis*. *CDF3* expression analyses using *promoter-GUS* fusions showed that *CDF3* is expressed in roots and root hairs, and in the perivascular tissues of root/stem and leaves in response to N starvation treatments (Figure 1), implying a possible function of this gene in root development. In fact, our results reveal that in contrast to the *cdf3-1* mutant, the overexpression of *CDF3* promotes increased LR and PR length under low nitrate conditions in vertical grow assays (Figure 3). The expression of genes involved in root development in response to N availability, such as *NRT2.1* (Little et al., 2005), changed significantly in the *CDF3*-overexpressing and knockout plants (Figure 4). However, we found no significant differences in LR density compared to the WT under both N conditions, which suggest that CDF3 could play a significant role in root elongation (Supplementary Figure S2). Overall, these data might indicate that CDF3 is involved in NO_3_^−^ modulation of root growth and development and overexpression of *CDF3* promotes root development.

### The Role of CDF3 in the Supply of C Skeletons for N Assimilation and Plant Growth

Nitrogen assimilation is closely interconnected to C metabolism, and plant growth relies on this interaction. Photosynthesis generates reducing power and C skeletons for the assimilation of nitrogen. It is well-known that PEPC1 and PK are key elements to fuel N assimilation machinery with carbon skeletons (Foyer et al., 2003). On this regard, plants have evolved complex mechanisms to sense and coordinate N assimilation with C metabolism and meet the demands required by growth and development (Stitt and Krapp, 1999; Sato et al., 2011; Sulpice et al., 2013). Previous results showed that maize DOF1 TF play a role in nitrogen assimilation through the control of the expression of *PEPC1* and *PK* genes (Yanagisawa, 2004). In order to investigate the role of the Dof transcription factor CDF3 in the control of the supply of C skeletons for the synthesis of N compounds, we analyzed the expression of *PEPC1* and *PK1* genes in *CDF3* overexpressor, *cdf3-1* and WT plants. Interestingly, we found that CDF3 behaves in a dual manner by controlling the expression of both genes depending on the N supply. In fact, we observed that under N non-limiting conditions, CDF3 behaves as a transcriptional activator of both *PEPC1* and *PK1* genes, facilitating N assimilation by feeding C skeletons and thus increased biomass production. In contrast, it plays a role as a repressor of both genes under N limiting conditions, likely to avoid the metabolic unbalance and accumulation C compounds like 2-OG, which could then not be used in N assimilation. Altogether, our results support an integrative dual regulatory role of CDF3 in C/N balance through the control of both N assimilation and C skeleton production genes depending on nitrogen availability (Figure 9). These results are in agreement with previous studies suggesting that *ZmDOF1* also controls C biosynthesis pathway depending on plant N status (Yanagisawa, 2004). However, protein sequence analyses show limited sequence similarity between both proteins, ZmDOF1 and CDF3, except in the DOF DNA binding domain (Supplementary Figure S7), suggesting that CDFs in dicot plant species could play similar functions in C/N metabolism as the ones displayed by *DOF1-like* genes in monocot species.

### *CDF3* Overexpression Enhances Tomato Biomass Production and N Accumulation Efficiency

The results obtained in this work support an important role of *Arabidopsis CDF3* in N assimilation during plant growth and in the response to changes in N availability. This led us to explore whether the functionality and effects observed in *Arabidopsis* are conserved in other plant species and it could be used to improve N use efficiency in a crop plant like tomato. We confirmed that *35S::CDF3* tomato plants exhibited higher CO_2_ fixation capacity and sugar content under optimal N fertilization. In addition, we showed that the overexpression of *CDF3* increased N assimilation, as inferred from the higher total N and amino acid contents (Figures 6, 8; Supplementary Table S2). Consistently, the *CDF3* overexpressing tomato plants showed increased biomass production, and notably, increased fruit yield and NAE.

In general, NUE can be divided into the components of N acquisition, assimilation, distribution, and utilization (Plett et al., 2017). The methodology used in this work (Weih, 2014; Weih et al., 2018) allowed a robust and reliable determination of the equivalent NAE parameter and the components related to the N acquisition and assimilation (N uptake efficiency), and utilization and distribution (yield-specific N efficiency and fruit yield N concentration). The higher overall N accumulation efficiency of the plants overexpressing *CDF3* gene under optimal N supply was due to increases in all three NAE components. Our data indicate that CDF3 improves N use efficiency under optimal N supply by enhancing the amounts of photoassimilates partitioned to the fruits, resulting from increased C and N metabolism, transport and sink strength of the tomato fruits. Moreover, we observed that under limited N condition, photosynthetic rate, biomass accumulation, and fruit yield were also enhanced in the *35S::CDF3* plants compared to controls (Figures 7, 8). Although a reduction was provoked by the limitation in N supply in control and *35S::CDF3* plants, photosynthetic rates, biomass accumulation, and yield were higher in the transgenic plants. Interestingly, under N limited supply, the N uptake efficiency did not differ between both genotypes. However, *35S::CDF3* plants maintained higher C/N supply to the fruits and in accordance higher yield under N limiting supply. These data support a key role of CDF3 in the regulation of photoassimilate partitioning to the fruits under both optimal and limiting N supply.

In conclusion, the present work, we identified *CDF3* as a new important regulatory factor of the nitrogen responses in *Arabidopsis* and tomato, linking key aspects of C and N metabolism, root development, and plant growth. Altogether, our results highlight *CDF3* as a new potential target to improve crop production in the context of sustainable agriculture that aims to improve crop production, while at the same time being environmentally sustainable, and ensuring healthy and productive soils for the future.

## Data Availability Statement

The raw data supporting the conclusions of this article will be made available by the authors, without undue reservation.

## Author Contributions

R-VM, SN, JV-C, and JM designed, planned, and organized the experiments. JD-F, LC, BR-M, LY, MW, and DM performed the research. R-VM, SN, JC, DM, and JM wrote the article. All authors contributed to the article and approved the submitted version.

### Conflict of Interest

The authors declare that the research was conducted in the absence of any commercial or financial relationships that could be construed as a potential conflict of interest.

## Footnotes

^1^http://bar.utoronto.ca/efp/cgi-bin/efpWeb.cgi
