# Supplementary material for: The Arabidopsis Transcription Factor CDF3 Is Involved in Nitrogen Responses and Improves Nitrogen Use Efficiency in Tomato
Source: Front Plant Sci. 2020 Nov 24;11:601558. doi: 10.3389/fpls.2020.601558 (PMC7732579; doi:10.3389/fpls.2020.601558)
Supplement: Supplementary file 1 [file Data_Sheet_1.pdf]

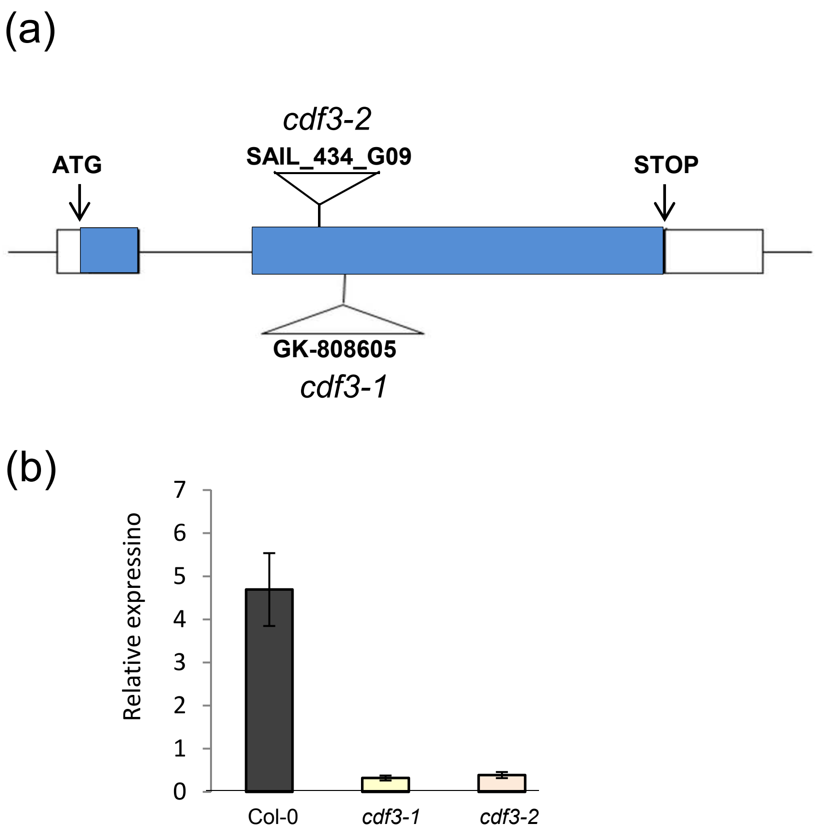

Figure S1. Genomic structure of CDF3 and localization of T-DNA in *cdf3* mutants.

(a) Scheme of the T-DNA insertion mutants *cdf3-1* GABI-Kat (GK-808605) and *cdf3-2* SAIL-434_G09. The T-DNA insertion in *cdf3-2* is located 641 pb from ATG. **(b)** *CDF3* gene expression analysis. Expression of *CDF3* gene was analysed by qRT-PCR in Col-0, *cdf3-1* and *cdf3-2* mutant plants. Total RNA was extracted from leaves of 3-week-old Arabidopsis plants.

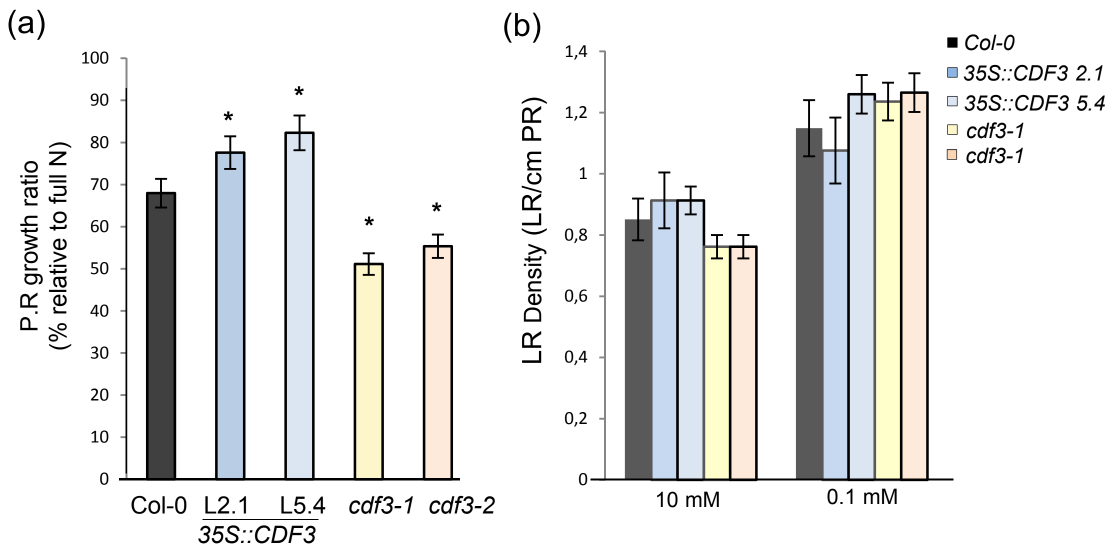

Figure S2. Root architecture analyses of *CDF3*-overexpressing, *cdf3-1, cdf3-2* and WT plants.

Plants were grown on vertical plates with 10 or 0.1 mM KNO3 as sole N source, for 12 days. (a) Primary root (PR) lengths were estimated under different nitrate conditions. Results are represented as percentage of root length under N-limiting (0.1 mM N) relative to nitrate rich conditions (10 mM N). (b) Lateral root density was estimated under different nitrate conditions. Lateral root density was estimated as the number of lateral root (LR) normalized by the primary root length (cm). Data are means ± SE of three independent experiments with at least 20 plants each. Asterisks indicate significant differences compared with wild type (Col-0) ($P<0.05$); ANOVA Student-Newman-Keuls tests

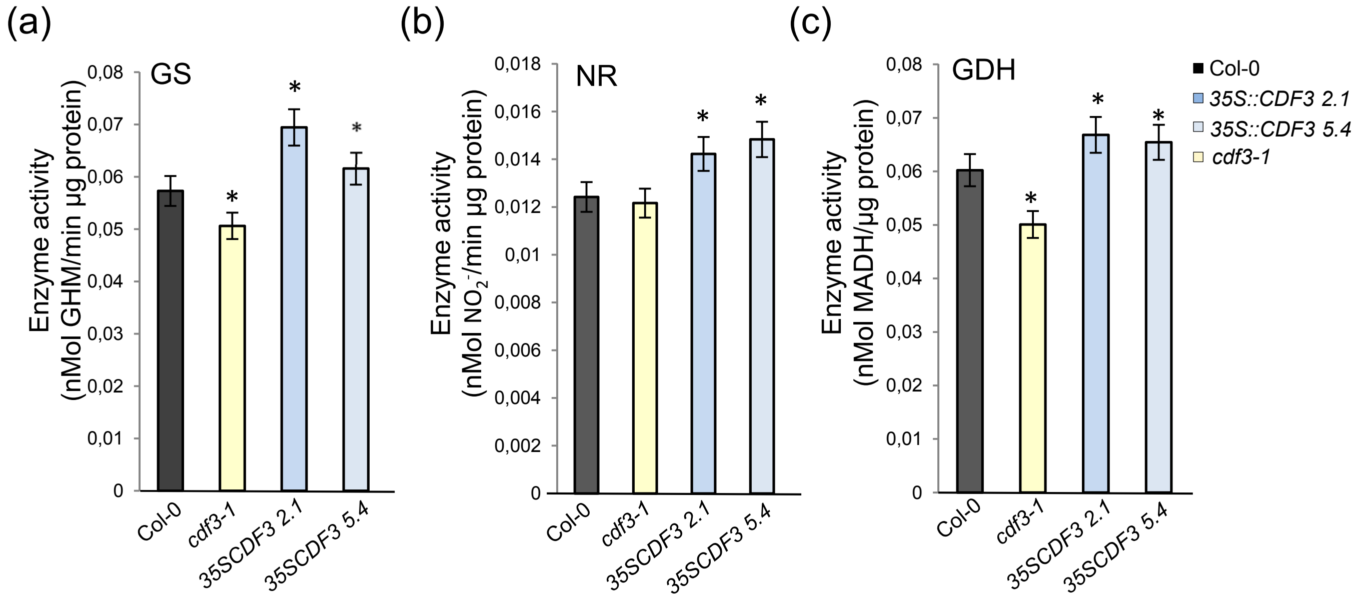

Figure S3. **Enzyme activities of Glutamine synthetase, Nitrate reductase, and Glutamate dehydrogenase in *CDF3* gain- and loss-of-function Arabidopsis lines**.

Enzyme activities **(a)** Glutamine synthetase (GS), **(b)** Nitrate reductase (NR), and **(c)** Glutamate dehydrogenase (GDH) were determined as described by Sarasketa *et al.*, (2014). Protein extracts form 12-day-old plants from Col-0, *cdf3-1* and *35S::CDF3* (L2.1 and L5.4) Arabidopsis lines were grown on MS medium containing 10 mM KNO_3 as sole nitrogen source. Asterisks indicate significant differences compared with wild type (Col-0) ($P<0.05$); ANOVA Student-Newman-Keuls tests.

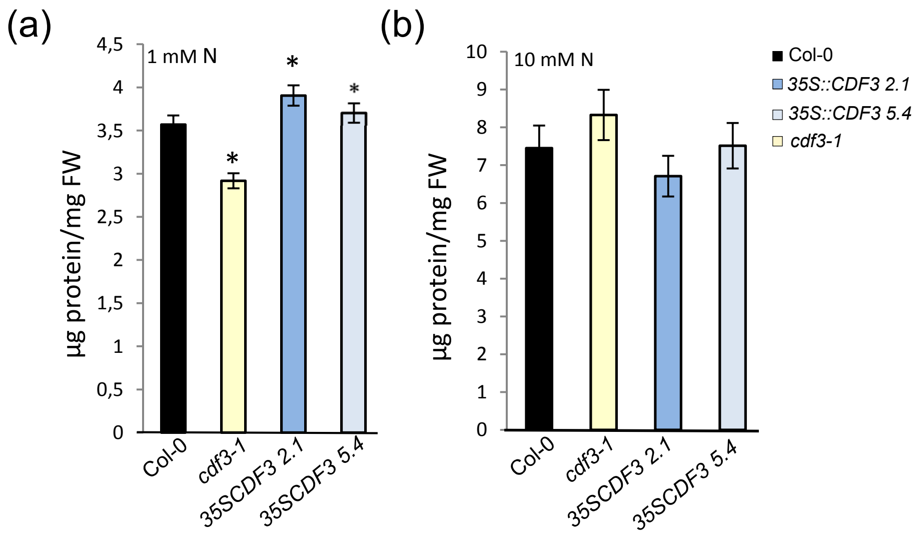

Figure S4. Protein content in *CDF3 gain- and loss-of-function* Arabidopsis lines.

Protein content was determined as described by Sarasketa *et al*., (2014). Protein extracts from 12-day-old plants from Col-0, *cdf3-1* and *35S::CDF3* (L2.1 and L5.4) Arabidopsis lines were grown on MS medium containing 1 or 10 mM KNO_3 as sole nitrogen source. Data are means ± SE of three independent experiments with at least 20 plants each. Asterisks indicate significant differences compared with wild type (Col-0) ($P<0.05$); ANOVA Student-Newman-Keuls tests.

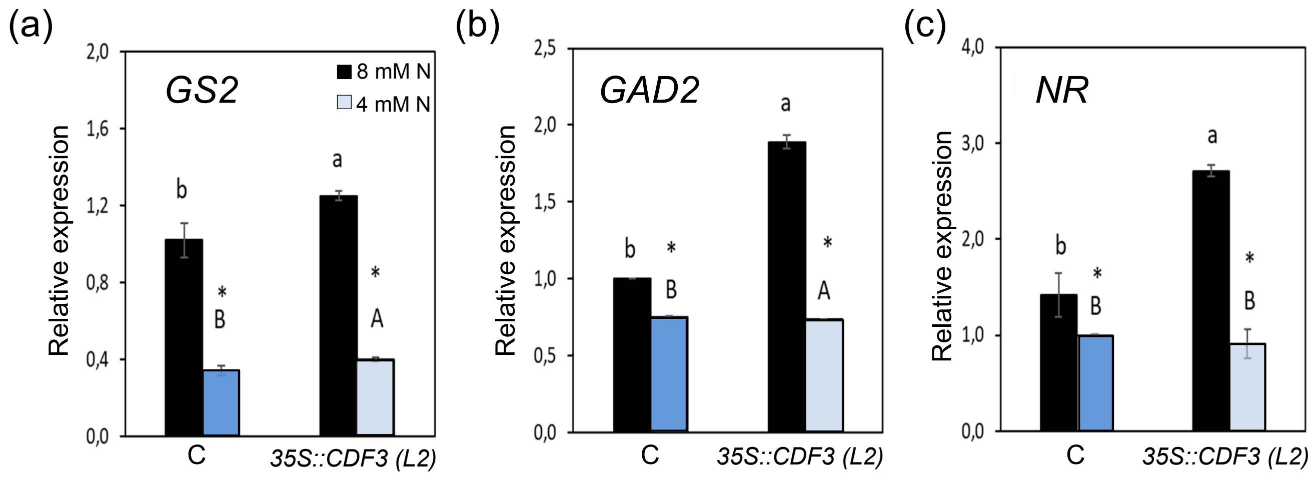

Figure S5. Expression analyses of tomato *Glutamate synthase (GS2), Glutamate decarboxylase (GAD2)* and *Nitrate reductase (NR) genes* in Moneymaker and *35S::CDF3* tomato plants.

Expression analyses by qRT-PCR *of* **(a)** *Glutamine synthetase 2 (GS2),* **(b)** *Glutamate decarboxylase 2 (GAD2) and* **(c)** *Nitrate reductase (NR) tomato genes* in Moneymaker cv (C) and *35S::CDF3* (L2) tomato plants. Total RNA was extracted from leaves of 55-day-old tomato plants grown in growth chamber conditions under 8mM and 4mM N nitrogen supply. Tomato *UBIQUITIN3* gene was used as a reference gene. Data are means ± SE (n=3). Different small or capital letters indicate significant differences between genotypes at 8 and 4 mM N, respectively (P<0.05). For each genotype, significant differences by the nitrogen supply are indicated by an asterisk (P<0.05). ANOVA Student-Newman-Keuls tests.

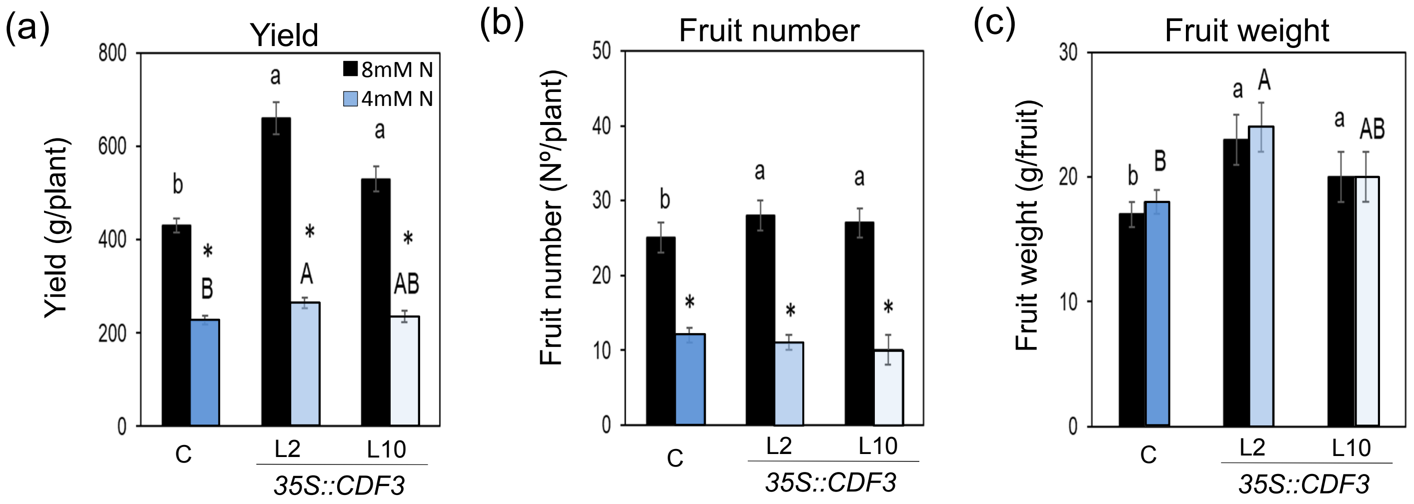

Figure S6. Increased yield in plants overexpressing the *CDF3* gene under both N non limiting and limiting conditions.

Total yield (g fruits/plant) **(a)**, number of fruits (no fruits/plant) **(b)** and mean fruit size **(c)** of the *35S::CDF3* plants (lines L2 and L10) and Moneymaker (C) grown in greenhouse conditions under 8mM N (black bars) and 4mM N (white bars) nitrogen supply. Each value is the mean (±SE) of ten different plants. Different small or capital letters indicate significant differences between genotypes at 8 and 4 mM N, respectively (P<0.05). For each genotype, significant differences by the nitrogen supply are indicated by an asterisk (P<0.05). ANOVA Student-Newman-Keuls tests.

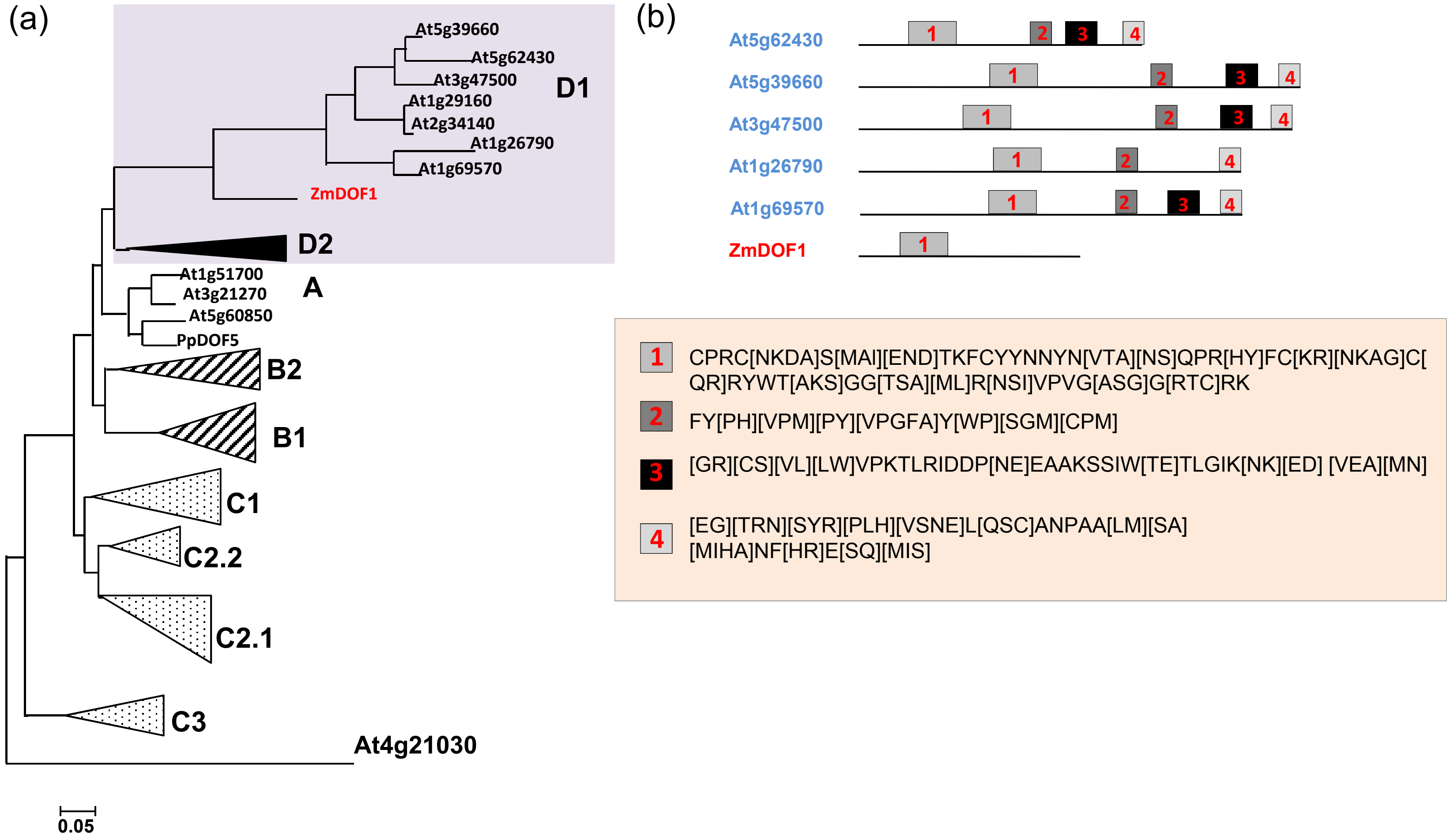

Figure S7. Phylogenetic trees and conserved motifs of *Arabidopsis* DOFs and Maize ZmDOF1 protein.

(a) *Arabidopsis* tree was inferred by the neighbour-joining method after alignment of the DOF domain amino acid sequences of the 36 *Arabidopsis* (Lijavetzky *et al.*, 2003) DOFs, Maize ZmDOF1. The resulting groups are shown as A, B, C, or D, and numbers indicate defined subgroups. Bar, 0.05 estimated amino acid substitutions per site. **(b)** Schematic distribution of conserved motifs among *Arabidopsis* CDF and Maize ZmDOF1. Motifs were identified by means by MEME software using the complete amino acid sequences of the DOF proteins clustered in group D of the phylogenetic trees. The position of the identified motifs is relative to the DOF domain. Multilevel consensus sequences for the MEME-defined motifs are listed.

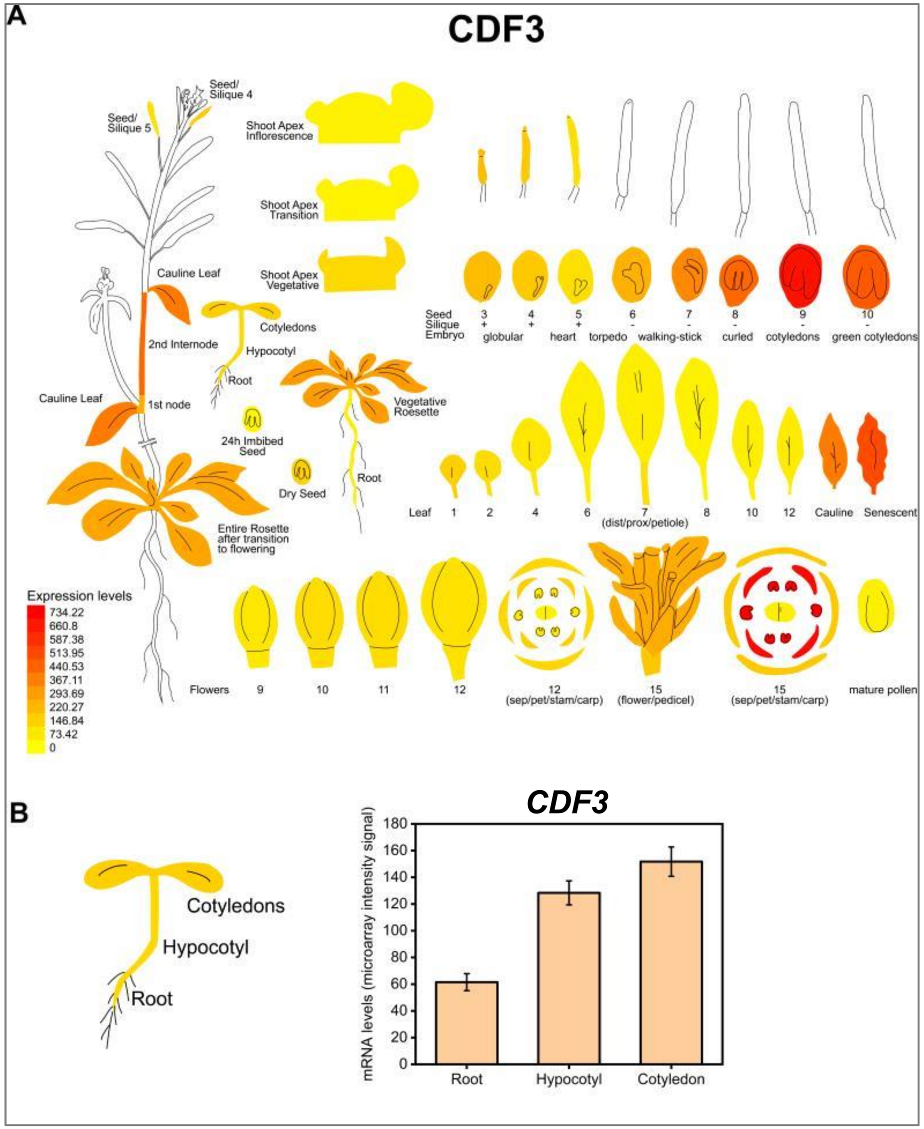

Figure S8. The expression pattern of *CDF3* in Arabidopsis.

(a) Spatial expression patterns of *CDF3* in Arabidopsis using the publicly accessible microarray database (eFP browser; http://bbc.botany. utoronto.ca/efp/ cgi-bin/efpWeb.cgi). (b) *CDF3* Expression patterns in seedlings.

Table S1. Primers used for Real-Time PCR analyses in Arabidopsis and tomato

Arabidopsis

Primer name	Sequence (5’-3’)
Ubi Fw	5´-GCTCTTATCAAAGGACCTTCGG-3`
Ubi Rv	5´-CGAACTTGAGGAGGTTGCAAAG-3´
AtCDF3 Fw	5´-AGAAGGCCGGGTGCGTTCTG-3´
AtCDF3 Rv	5´-ACCGGCTTTGCACATCGCCT-3´
GLU1 Fw	5´-CTATGGAGAGCAGATTAATGG-3´
GLU1 Rv	5´-GTACAGTTATAGCAACCATGG-3´
GS1.1 Fw	5´-CAACCTTAACCTCTCAGACTCCACT-3´
GS1.1 Rv	5´-CAGCTGCAACATCAGGGTTGCTA-3´
GS1.4 Fw	5´-CAATCTCGATCTCTCCGATTCCACT-3´
GS1.4 Rv	5´-GGCGACAACACTAGGGTCTTCA-3´
GS2 Fw	5´-GAATCTGATGAACACACGTGTC-3´
GS2 Rv	5´-GGACATGCTCTAACAGTC-3´
ASN1 Fw	5´-TGACGACTATCAGAGCGAGCAC-3´
ASN1 Rv	5´-ACTTGTGAAGAGCCTTGATCTTGC-3´
NIA1 Fw	5´-CTGAGCTGGCAAATTCCGAAGC-3´
NIA1 Rv	5´-CCCTCGTTACCTTCTTACCTCCTC-3´
NRT2.1 Fw	5´-AATCGAGCTACCTTGGAGAAAGC-3´
NRT2.1 Rv	5´-GTGGAGCTTCAAGTGAAACCTGTC-3´
NRT2.4 Fw	5´-CCGTCTTCTCCATGTCTTTC-3´
NRT2.4 Rv	5´-CTGACCATTGAACATTGTGC-3´
NRT2.5 Fw	5´-CTCCTCCCTGTTATCCGTGAAA-3´
NRT2.5 Rv	5´-AGACGAAAGTGGCGAGAGAGAA-3´
PK1 Fw	5´-CGTCAGGCCTCCTCCTTCATTC-3´
PK1 Rv	5´-GTTCATGTGTCAGAAGG-3´
PEPC1 Fw	5´-CTTTGAATCTCTCTTTCTCTCTC-3´
PEPC1 Rv	5´-CTCGAAGTACTCGTACACG-3´
ATL31 Fw	5´-TTGCTGAACAGACGCCTGAACC-3´
ATL31 Rv	5´-AAGCGTAAACCGGTCGGTACTC-3´
WRKY53 Fw	5´-ACAGAGGAACACACACTTGTTCGC-3´
WRKY53 Rv	5´-ACCATCATCAAGCCCATCGGTTC-3´

Tomato

Primer name	Sequence (5’-3’)
NR Fw	5’-CGGTTCGTGGTTGCAACTTC-3’
NR Rv	5’-CCAATTATCAGCGGTACCTTC-3’
GAD2 Fw	5’-CGTCGTTGTACCACCACTACGC-3’
GAD2 Rv	5’-ACGCGAAAGTCGAGTGAACGG-3’
GS2 Fw	5’-AGCTTCAGCCTCAAGGGTTGGC-3’
GS2 Rv	5’-CGCCCAGCTTCAAACATGGACC-3’
UBI3 Fw	5’-AAGCAATGGATGCTGAGGCT-3’
UBI3 Rv	5’-GAAGGTGCCGTTGAATGACA-3’

Table S2. Photosynthetic and carbon metabolism parameters in *35S::CDF3* tomato plants under non-limiting (8 mM N) and limiting (4 mM N) nitrogen supply. Values are mean of 10 different plants. Measurements and determinations were performed in the 3-4 leaf from the apex in plants maintained during 25 days under differential N supply. Stomatal conductance (g_s) and effective PSII quantum yield (PhiPS2) are mean of ten measures in different plants. Total soluble sugars, starch and total α amino acid values are mean of 4 different determinations.

Nitrogen supply	Genotype	g_s (mol / m^2s)	PhiPS2	Soluble sugars (µg/ mg DW)	Starch (µg/ mg DW)	α-amino acids (µmol/ mg DW)
8 mM N	C	0.25 b	0.077 b	43 b	27	217 b
	35S::CDF3 L2	0.35 a	0.097 a	55 a	29	235 a
	35S::CDF3 L10	0.37 a	0.095 a	52 a	28 $_{NS}$	241 a
4 mM N	C	0.15 b	0.055 b	57 b	32 b	196
	35S::CDF3 L2	0.25 a	0.078 a	64 a	40 a	179
	35S::CDF3 L10	0.22 a	0.074 a	60 a	37 a	189 $_{NS}$

For each parameter and nitrogen level, different letters indicate significant differences (P<0.05). NS: not significant

Appendix S1. Methods used for NAE determinations.

The N accumulation efficiency (NAE) and its components were assessed in tomato according to the methodology by Weih M. (2014) and Weith *et al.,* (2018). This approach integrates ecological, agricultural and physiological concepts, considering the whole life cycle of the crop. Thus, the N accumulation efficiency is decomposed into three components: N uptake efficiency (U_N; g/g) as the ratio between mean plant N content during the main growth period and N in the seed; yield-specific N efficiency ($E_{N,y}$; g/g) as the ratio between fruit yield and the mean plant-internal N content during the main growth period; and fruit yield N concentration ($C_{N,y}$; g/g). Accordingly, the overall NAE is the final N yield divided by the N content in the initial plant material, and thus the ability of crops to multiply the N available in the initial seed; and NAE = U_N x $E_{N,y}$ x $C_{N,y}$.

The three components are calculated based on the biomass and N pools determined at the seed, intermediate (starting flowering period) and final plant (fruit production) stages. Thus, tomato seeds and harvested plants were oven-dried at 70ºC for 48 h and weighed. The N contents of seeds and plant organs (roots, stems, leaves and fruits) were determined at the CEBAS-CSIC laboratory of ionomics (CEBAS, Murcia, Spain) using a CHN elemental analyzer. Finally, the three NAE components were calculated according to the protocol by Weih (2014).

References

Weih M. (2014). A calculation tool for analyzing nitrogen use efficiency in annual and perennial crops. *Agronomy,* 4: 470-477.

Weih, M., Hamnér, K. & Pourazari, F. (2018). Analyzing plant nutrient uptake and utilization efficiencies: comparison between crops and approaches. *Plant Soil* **430,** 7–21
